# Mold infections in chronic granulomatous disease patients—what comes to the rescue?

**DOI:** 10.1038/s41409-024-02342-y

**Published:** 2024-11-04

**Authors:** Horst von Bernuth, Tayfun Güngör

**Affiliations:** 1 Department of Pediatric Respiratory Medicine, Immunology and Critical Care Medicine, Charité - Universitätsmedizin Berlin, corporate member of Freie Universität Berlin, Humboldt-Universität zu Berlin, and Berlin Institute of Health, Berlin, Germany; 2 Division of Hematology/Oncology/Immunology, Gene-therapy, and Stem Cell Transplantation, University Children’s Hospital Zurich – Eleonore Foundation & Children’s Research Center (CRC), Zurich, Switzerland

**Keywords:** Immunological deficiency syndromes, Risk factors

In this issue of *Bone Marrow Transplantation*, Kline et al. present 26 patients with Chronic Granulomatous Disease (CGD) who had stable (*n* = 8), progressive (*n* = 13), or incident (*n* = 5) mold infections treated with cellular therapies (CT), among others, to cure the underlying and, in 21 patients, intractable fungal disease [1]. This is the largest such report focussing on patients with CGD and mold infection undergoing CT [1]. The median age at time of receipt of allogeneic hematopoietic cell transplantation (HCT) or autologous gene therapy-modified cells (GT) was 18 years (IQR 11–27). The median duration of fungal infection was 1.75 years (IQR 1–6 years) indicating chronic infections. 5 patients had incident mold disease, not diagnosed prior to CT, rendering this group different from the other 21 patients. Before the application of CT, 20 patients presented mainly with pneumonia, 9 of them with osteomyelitis due to direct spread. Three patients had pre-existing central nervous system (CNS) mold infection (2 incidental and one, patient 8, “stable then incidental”). In all patients, except for one, mold was isolated (*Scedosporium apiospermun*, *Aspergillus*, *Phellinus*, *Rhizopus* spp.) and antifungal susceptibilities were available. Although the exposure of patients with dysfunctional phagocytes and mold infection to chemotherapy-induced neutropenia, tissue damage, and consecutive release of pro-inflammatory cytokines seems contradictory, the engraftment of functional phagocytes contributes to control and cure of mold infection in patients with CGD. This proof of principle had been shown already in 1998 in a child cured of disseminated *Aspergillus nidulans* infection by Hematopoietic Stem Cell Transplantation (HSCT) [2] and has been demonstrated in subsequent case series and in several patients enrolled in a prospective study of HCT in patients with CGD [3–6].

CGD is a heterogeneous inborn deficiency of the phagocytic NADPH-oxidase-complex, causing critically reduced production of reactive oxygen intermediates upon phagocytosis of bacteria and fungi. Reactive oxygen intermediates are required to kill intracellular pathogens and to abrogate pro-inflammatory processes [7]. Among fungal infections, *Aspergillus* spp are the most often encountered in patients with CGD, but others (e.g., *Scedosporium* spp, *Phellinus* spp) have been identified [2–6]. In general, patients with CGD have a ~40% lifetime risk for mold infections. However, compensatory killing mechanisms do exist in phagocytes in the absence of NADPH oxidase, explaining that ~60% of individuals with CGD who never develop invasive mold infections despite ubiquitous daily airborne exposure to i.e., *Aspergillus conidia*. In addition, patients with CGD rarely develop infection by the ubiquitous molds Rhizopus or Fusarium species, indicating that these fungi can be effectively controlled by non-oxidative cytotoxic mechanisms [8]. The introduction of life-long antibacterial and antifungal prophylaxis with azoles is mandatory for patients with CGD and has improved overall survival substantially. Nevertheless, breakthrough fungal infections which are progressive despite antifungal prophylaxis remain a substantial cause of morbidity and death in patients with CGD [3, 4, 6].

Mold infections in CGD differ from those in secondary neutropenia due to chemotherapy [8, 9]. Granulomas in CGD consist of spherical structures with a central core of tissue-resident epithelioid macrophages which merge into multinucleated giant cells surrounded by T cells. Mold infection in CGD patients can also lead to chronic necrotizing granulomatous inflammation, which is a semi-invasive form of mold infection. Here, fungal hyphae are usually identified within the areas of tissue necrosis and parenchymal invasion is apparent, but vascular invasion is not [9]. The histopathology shown by Kline et al. demonstrates mainly granulomatous inflammation, including neutrophilic abscess formation, epithelioid histiocytes, and multinucleated giant cells surrounding fungus in patients with CGD [1]. These granulomatous patterns are histologically distinct from fungal lesions in patients with chemotherapy-induced neutropenia, which are mainly characterized by central pale necrotic zones, surrounded by a hemorrhagic rim, necrotic debris, and tissue/vascular invasion by fungal hyphae. In chemotherapy-induced neutropenia, this leads to vessel occlusion, localized infarction, and vascular erosion with risk of arterial hemorrhage [9]. The distinct pattern of granuloma formation in CGD with usually non-occluded vessels, surrounding invasive molds may facilitate functionally corrected phagocytes after CT to penetrate fungal granulomas.

Kline et al. demonstrate cures of invasive mold infections in patients with CGD upon CT [1]. Their report also implies, that once mold infections are established in CGD, they are mostly intractable to management by antifungals alone [1–6]. That after CT functionally normalized phagocytes are capable to produce sufficient amounts of reactive oxygen intermediates and that granulocyte infusions are dispensable is another important confirmatory message [5, 6]. Pulmonary and cerebral hemorrhages accounted for 5 deaths (2 pulmonary, and 3 CNS) after CT, with the caveat that in 2 of them, severe post CT-complications (i.e., ITP and Evans syndrome) probably contributed to bleedings. This highlights that mold infections in the CNS of CGD patients are difficult to treat [5]. Since pro-inflammatory cytokines are strongly increased in tissues of CGD-patients exaggerated leucocyte influx is to be expected after engraftment. Even though anti-inflammatory agents like Tocilizumab were used, we believe that in addition to in-vivo T-cell depletion early steroid co-medication is beneficial in infected CGD patients to prevent engraftment syndrome. We also believe that peripheral blood stem cells (PBSC) should be used with caution in CGD transplantation, as PBSC contains an increased concentration of mature T-cells by about one log [10]. A limitation of the study is the heterogeneity of conditioning regimens, HCT donor sources, and cell therapy type (HCT and GT). This makes it impossible to assess details of each therapeutic approach and its contribution to the success of the CT and the intended cure of the underlying mold infections [1].

Pre-treating CGD patients with mold infections prior to CT until the inflammation is substantially diminished seems reasonable. We pre-treat patients at least 4 weeks with intravenous antifungals according to susceptibility testing and add steroids once sufficient trough levels of azoles are achieved. Azole treatment profoundly interacts with drugs used for conditioning, mainly busulfan. Therefore, therapeutic drug monitoring for busulfan, as performed here, is mandatory under continued azole treatment for mold infections. Shortening the time of severe mucositis and neutropenia prevents spread or progression of molds. In the present work by Kline et al., the median duration of neutropenia was approximately two weeks (IQR 10–21 days). There was a roughly 30% mortality during the observation period, which is notably higher than that reported HCT for patients with CGD without mold infection [10, 11]. Nevertheless, with the exception for patients with incident CNS mold infection, CT offers a reasonable option for patients with CGD who have been treated unsuccessfully for fungal infection for months or even years [1–6]. In CGD transplantation, deaths are usually transplantation-associated (mainly graft failure, graft versus host disease (GVHD), and secondary autoimmunity) and not due to underlying infections [3, 5, 10, 11]. This is confirmed here, with the exception of the patient with *Aspergillus felis* infection in whom the disease progressed despite adequate neutrophil engraftment and myeloid chimerism. The work by Kline et al. further confirms that outcomes after CT, especially after HSCT, have improved significantly over the last decades, particularly when Human Leukocyte Antigen (HLA)-identical donors are available [5, 10, 11]. GT for CGD has also shown functional correction of phagocytes, but graft failures are not uncommon [12].

The overall survival in CGD patients after CT is encouraging [10, 11]. As such, patients with newly diagnosed CGD are often offered curative CT and less frequently develop mold infections. However, CGD patients remain who will benefit from the more detailed knowledge that only CT is curative for mold infections in CGD. Among these are young adults and their families who had been advised that “in CGD HSCT is too risky”. A second group may include patients whose parents prefer continuing on conservative therapy. These will become the adult CGD patients of tomorrow. The current study by Kline et al. adds to the encouraging reports of curative CT of CGD in adolescents and adults. In CGD chronic fungal infections are an indication to proceed to CT.
